# Development and Performance of a Clinical Decision Support Tool to Inform Resource Utilization for Elective Operations

**DOI:** 10.1001/jamanetworkopen.2020.23547

**Published:** 2020-11-02

**Authors:** Benjamin A. Goldstein, Marcelo Cerullo, Vijay Krishnamoorthy, Jeanna Blitz, Leila Mureebe, Wendy Webster, Felicia Dunston, Andrew Stirling, Jennifer Gagnon, Charles D. Scales

**Affiliations:** 1 Department of Biostatistics and Bioinformatics, Duke University, Durham, North Carolina; 2 Department of Population Health Sciences, Duke University, Durham, North Carolina; 3 Surgical Center for Outcomes Research, Duke University, Durham, North Carolina; 4 Duke Clinical Research Institute, Duke University, Durham, North Carolina; 5 Department of Surgery, Duke University, Durham, North Carolina; 6 Department of Anesthesiology, Duke University, Durham, North Carolina; 7 Critical Care and Perioperative Population Health Research Unit, Duke University, Durham, North Carolina; 8 Department of Neurosurgery, Duke University, Durham, North Carolina; 9 Department of Head & Neck Surgery and Communication Sciences, Duke University, Durham, North Carolina; 10 Duke Health Technology Solutions, Duke University Health System, Durham, North Carolina

## Abstract

**Importance:**

Hospitals ceased most elective procedures during the height of coronavirus disease 2019 (COVID-19) infections. As hospitals begin to recommence elective procedures, it is necessary to have a means to assess how resource intensive a given case may be.

**Objective:**

To evaluate the development and performance of a clinical decision support tool to inform resource utilization for elective procedures.

**Design, Setting, and Participants:**

In this prognostic study, predictive modeling was used on retrospective electronic health records data from a large academic health system comprising 1 tertiary care hospital and 2 community hospitals of patients undergoing scheduled elective procedures from January 1, 2017, to March 1, 2020. Electronic health records data on case type, patient demographic characteristics, service utilization history, comorbidities, and medications were and abstracted and analyzed. Data were analyzed from April to June 2020.

**Main Outcomes and Measures:**

Predicitons of hospital length of stay, intensive care unit length of stay, need for mechanical ventilation, and need to be discharged to a skilled nursing facility. These predictions were generated using the random forests algorithm. Predicted probabilities were turned into risk classifications designed to give assessments of resource utilization risk.

**Results:**

Data from the electronic health records of 42 199 patients from 3 hospitals were abstracted for analysis. The median length of stay was 2.3 days (range, 1.3-4.2 days), 6416 patients (15.2%) were admitted to the intensive care unit, 1624 (3.8%) received mechanical ventilation, and 2843 (6.7%) were discharged to a skilled nursing facility. Predictive performance was strong with an area under the receiver operator characteristic ranging from 0.76 to 0.93. Sensitivity of the high-risk and medium-risk groupings was set at 95%. The negative predictive value of the low-risk grouping was 99%. We integrated the models into a daily refreshing Tableau dashboard to guide decision-making.

**Conclusions and Relevance:**

The clinical decision support tool is currently being used by surgical leadership to inform case scheduling. This work shows the importance of a learning health care environment in surgical care, using quantitative modeling to guide decision-making.

## Key Points

**Question:**

How can clinical departments implement a clinical decision support tool to predict expected resource use to prioritize elective inpatient surgical procedures?

**Findings:**

In this prognostic study, predictive models for length of stay, intensive care unit length of stay, mechanical ventilator requirement, and discharge disposition to a skilled nursing facility were developed using historical case data abstracted from the electronic health records of 42 199 patients. These models were integrated into an interactive online dashboard with end-user input and iteratively tested.

**Meaning:**

Predictive modeling, in conjunction with other contextualizing factors, can be used to inform how to recommence elective inpatient procedures after the coronavirus disease 2019 (COVID-19) pandemic.

## Teaser

In this prognostic study, the development and performance of a clinical decision support tool to inform resource utilization for elective procedures requiring inpatient care were evaluated.

## Introduction

The novel coronavirus disease 2019 (COVID-19) has changed the provision of hospital- and clinic-based surgical care. As hospitals prepared for possible surges of infected patients requiring admission and possible intensive care (ICU) stay, entire institutions and health systems took stock of their resources to meet an uncertain demand. This included estimating an ever-fluctuating number of available beds, securing sufficient personal protective equipment and ventilators, minimizing staff shortages, and establishing protocols to mitigate against nosocomial infections. National specialty societies examined the lessons from their counterparts abroad^1,2,3^ and, along with federal and state agencies, issued guidelines for the evaluation of the urgency of procedures.^4,5,6^

Consequently, during the initial wave of infections starting in March of 2020, many hospitals—including our own—severely restricted elective procedures. Although few operations are optional, some are more urgent than others. This includes procedures such as joint arthroplasties, plastic and reconstructive operations, and even certain oncologic resections. Examples of procedures that were not included would be emergency procedures, such as laparotomy for penetrating trauma, and urgent cardiovascular interventions for patients already admitted to the hospital. National specialty societies published guidelines for determining how to selectively postpone operations without compromising outcomes.^7,8^ As this initial wave of COVID-19 infections slows in our region, we are beginning to address the backlog of operations that have been postponed and are starting to perform these procedures once more. Even still, we must contend with the potential for local surges in cases and a renewed demand for hospital resources.

With input from health system leaders and experts in infection control, our surgical leadership developed 4 principal questions that, taken together, could help determine how and when an elective inpatient operation could be performed. So, for surgical patients: (1) How long would they stay in the hospital? (2) Would they require an ICU stay and for how long? (3) Would they need a ventilator? (4) Were they likely to be discharged to a skilled nursing facility (SNF)?

These questions were modular, applicable across specialties, and represented the individual hurdles to assessing perioperative patient flow. While these questions are not the only ones necessary to consider when deciding whether to perform a case—issues around medical need, overall backlog, and revenue are factored in—they provide a basis for understanding the real consequences of these scheduling decisions with regard resource use during the pandemic.

While clinical decision support (CDS) has permeated medical care, there are few surgical CDS tools to predict resource utilization. Most work has been specialty and/or procedure specific^9^ or has addressed questions of comparative effectiveness.^10,11^ The role of predictive analytics in improving operating room efficiency has been touted for day-of-staffing and resource-allocation decisions.^12,13^ However, there remains a need for a CDS that could be used across all specialties to evaluate the demand for resources imposed by increasing case volumes.

In this prognostic study, we present both our process for developing a predictive model for each of these constraints and the platform across which this tool has been implemented and accessed. The platform enables surgeons to evaluate a current schedule of cases, their clinical characteristics, and the predicted resources required to perform them. This CDS enables surgical leadership to assess the imminent caseload, incorporate additional data on current hospital resource use, and determine whether a case can proceed or be postponed. Our overall study question was to see whether we could use historic data to develop a CDS tool for resource utilization applicable during the COVID-19 pandemic.

## Methods

We followed the Transparent Reporting of a Multivariable Prediction Model for Individual Prognosis or Diagnosis (TRIPOD) reporting guideline for thedevelopment of predictive models. This study was declared exempt by the Duke University Health System (DUHS) institutional review board, and informed consent was waived, because the data were deidentified.

### Environment

In March 2020, the DUHS curtailed elective operations owing to concerns regarding the spread of COVID-19. By the end of May, the DUHS recommenced these procedures on a more limited basis. The DUHS consists of 3 hospitals—Duke University Hospital, a tertiary care and level 1 trauma center (939 total beds and 88 ICU beds)—and 2 community hospitals—Duke Regional Hospital (295 total beds and 22 ICU beds) and Duke Raleigh Hospital (185 total beds and 15 ICU beds). Since 2014, the DUHS has used a common electronic health record (EHR) for inpatient and outpatient reviewing of medical records; the ordering of laboratory tests, medications, and radiology studies; and operating room scheduling using the Epic platform.

### Stakeholder Engagement

At initiation, we engaged stakeholders representing key constituencies, including clinical operations, anesthesiology, critical care, case management, surgeons, and hospital or health system leadership. Weekly meetings included focused discussions to identify and refine model inputs and outputs with stakeholders. Hospital leadership stakeholders provided periodic feedback regarding outputs and the user interface. This engagement process strongly informed the selection of clinically and operationally impactful CDS outputs.

### Data

#### Case Definition

We abstracted EHR data on patients who underwent an elective inpatient procedure performed at DUHS hospitals from January 1, 2017, to March 1, 2020. While there is no formal designation for elective procedures within the EHR system, we included all procedures with the admission source of “Surgery Admit Inpatient.” This is a classification for procedures in which a patient is admitted following the surgical intervention for inpatient postoperative care and is not admitted via the emergency department. We further excluded any procedures performed on Saturday or Sunday. Procedures performed in an ambulatory setting were not included because they are rarely associated with inpatient resource utilization.

#### Definition of Outcomes

We sought to predict 4 outcomes for each patient: overall hospital length of stay (LOS), ICU LOS, mechanical ventilator requirement, and discharge to an SNF. The LOS was determined as the total number of hours a patient spent in the hospital. The ICU LOS was determined as the total number of hours a patient was boarded in an ICU (including nonconsecutive days). Because the LOS often has an extreme rightward skew,^14,15,16^ both the LOS and the ICU LOS were categorized into clinically meaningful ordinal groups with the input of critical care experts (eFigure 1 in the Supplement). The LOS was categorized into fewer than 2 days, 2 to fewer than 4 days, 4 to fewer than 7 days, and 7 days or longer. The ICU LOS was grouped into 0 days, fewer than 2 days, and 2 days or more.

#### Definition of Predictors

We abstracted patient- and procedure-specific data that would be known prior to the procedure date. These included demographic characteristics, medication history, comorbidities, and service utilization history (detailed in eTable 1 in the Supplement). Variables with high rates of incompleteness (eg, laboratory values) were excluded, totaling 44 unique predictors.

### Statistical Analysis

#### Development of Predictive Model

We randomly divided the data into training and testing sets (two-thirds and one-third, respectively). We considered different analytic methods, including regularized regression and ordinal regression, and ultimately decided on a random forest algorithm because of its performance on the training data and its suitability for the analytic task (eAppendix in the Supplement).^17^

We developed 4 separate predictive models (one for each outcome defined). Each random forest was grown to 2000 trees. For LOS, we created a multiclass model, predicting the probability of being in each of the 4 classes described. For ICU LOS, we used a 2-stage approach.^18,19,20^ First, we modeled the need for an ICU stay (yes or no) in a binary classification model. Then, among operations that resulted in a need for an ICU stay, we modeled a second binary classification model, which was used to identify an ICU LOS of fewer than 2 days (short) and of 2 or more days. Binary classification models were constructed to predict the need for a ventilator and for discharge disposition (SNF).

For each binary classification model (the 2-part ICU LOS model, need for ventilator, and SNF discharge disposition), we evaluated overall model performance using the area under the receiver operating characteristic (AUROC), the area under the precision recall curve (AUPRC), and the calibration slope in the cross-validated training data and independent test data. We evaluated the LOS model using cross-entropy loss and missclassification loss.

#### Creation of a CDS Rule

To facilitate decision-making, we generated predicted labels as opposed to predicted probabilities for each outcome, having found previously that such groupings are more interpretable to users.^21^ There are a variety of ways that cutpoints can be generated. We focused on optimizing the sensitivity (percentage of true events captured) of our classifications to mitigate concerns about exceeding available hospital capacity for postoperative care. Therefore, these classifications prioritized overestimation—rather than underestimation—of risk. The eAppendix in the Supplement details how we set model classification thresholds. In brief, we created the high- or medium-risk group to have a sensitivity of 95% (ie, most events would be in the higher-risk groups). Moreover, to engender confidence in the proposed classifications, we sought to optimize the positive predictive value—the percentage of patients in high-risk the group who will truly have the event. All cutpoints were created based on the cross-validated training data, and then the performance of these cutpoints was tested in the prespecified test set to evaluate model performance.

Statistical analyses were conducted in R version 3.6. The random forest model was fit with the ranger package.^22^

#### Visualization Aid

To aid decision support, we integrated the predictive models into a Tableau dashboard. Tableau is an interactive visualization tool, and we have described how we have integrated it with EHR data previously.^23^ Each morning by 6 am, scheduled cases for the next 30 days are extracted into a data table with the necessary clinical information to run the models. Our analytic script queries this table, generates predictions, and feeds them into the dashboard with other clinical information. We created 3 types of visualizations: an overall summary, a calendar interface for each outcome, and a model monitoring view. This allows surgeons and all coordinators of perioperative services to view both the predictors and the predicted outcomes for individual cases (ie, at the patient level), in the context of other upcoming scheduled cases.

## Results

### Descriptive Statistics

From January 1, 2017, to March 1, 2020, we identified 42 199 elective surgical procedures across our 3 hospitals. Table 1 presents descriptive statistics across the 3 hospitals. The median age of the patients was 62 years (range, 49-71 years); 22 321 of 42 199patients (52.9%) were female. As expected, patients at our community hospitals had a higher burden of chronic disease.^24^ The 5 most commonly performed procedures were knee arthoplasty (n = 3539), hip arthoplasty (n = 3263), shoulder reconstruction (n = 1227), cervical diskectomy (n = 1162), and microsurgery (n = 1112), accounting for 10 128 of all 42 199 procedures (24.0%). The median LOS was 2.3 days (range, 1.3-4.2 days), with 9.8% of patients having a LOS longer than 7 days (eFigure 1 in the Supplement). Of the 42 199 procedures, 6416 (15.2%) involved an ICU stay, with 3208 (5.0%) of the 6416 patients requiring more than 2 days in the ICU. In addition, of the 42 199 patients, 1624 (3.8%) required a ventilator, and 2842 (6.7%) were discharged to an SNF.

**Table 1.** Descriptive Statistics of Overall Test and Training Data

Characteristic	Patients, No. (%)	
	Academic center	Community centers
Total No. of cases	25 554	16 645
Age, median (IQR), y	62 (47-70)	63 (52-71)
Female sex	12 563 (49.1)	9758 (58.6)
Race/ethnicity		
Non-Hispanic White	18 560 (72.7)	12 194 (73.3)
Non-Hispanic Black	4764 (18.6)	3513 (21.1)
Hispanic	699 (2.7)	294 (1.8)
Other	1521 (1.8)	644 (3.9)
BMI category		
Normal	6364 (24.9)	3002 (18.0)
Overweight	7973 (31.2)	4831 (29.0)
Obese	9512 (37.2)	8689 (52.2)
Underweight	1662 (6.5)	118 (0.7)
Missing	43 (0.2)	5 (<0.1)
Comorbidities		
Diabetes	3953 (15.5)	3034 (18.2)
Chronic obstructive pulmonary disease	1757 (6.9)	1019 (6.1)
Congestive hear failure	1069 (4.2)	494 (3.0)
Myocardial infarction	210 (0.8)	111 (0.7)
Hypertension	10 011 (39.2)	7856 (47.2)
Peripheral vascular disease	1024 (4.0)	498 (3.0)
CVA (TIA)	645 (2.5)	371 (2.2)
Atrial fibrillation	1552 (6.1)	848 (5.1)
Atherosclerotic cardiovascular disease	2256 (12.7)	1870 (11.2)
Coronary artery disease	2908 (11.4)	1673 (10.1)
Cardiovascular disease	14 811 (58.0)	9758 (58.6)
Diabetic renal	875 (3.4)	502 (3.0)
End-stage kidney disease	395 (1.5)	114 (0.7)
Pulmonary hypertension	487 (1.9)	259 (1.6)
Stent	1397 (5.5)	330 (2.0)
Cardiac surgery	438 (1.7)	101 (0.6)
Service utilization history		
Ambulatory visits, median (IQR), No.	11 (6-22)	11 (6-21)
Any inpatient visits	5555 (21.7)	2323 (13.9)
Any emergency visits	3089 (12.1)	1770 (10.6)
Outcomes		
Length of stay, median (IQR), d	2 (1-4)	2 (1-3)
ICU stay	5412 (21.1)	1004 (6.0)
Need for ventilator	1413 (5.5)	211 (1.3)
Discharged to SNF	1534 (6.0)	1308 (7.9)

Abbreviations: BMI, body mass index; CVA (TIA), cerebrovascular accident (transient ischemic attack); ICU, intensive care unit; IQR, interquartile range; SNF, skilled nursing facility.

### Predictive Modeling Results

After dividing into training (n = 28 130) and testing (n = 14 069) sets, we found that the overall predictive performance was quite strong in the training (based on cross-validation) and testing data and generally well calibrated (eTable 4 in the Supplement). The strongest models were found for predicting need for ICU (AUROC = 0.94) and need for ventilator (AUROC = 0.92). Predictions were strong but a bit worse for discharge to an SNF (AUROC = 0.84) and long vs short ICU stay (AUROC = 0.76).

**Figure 1.**
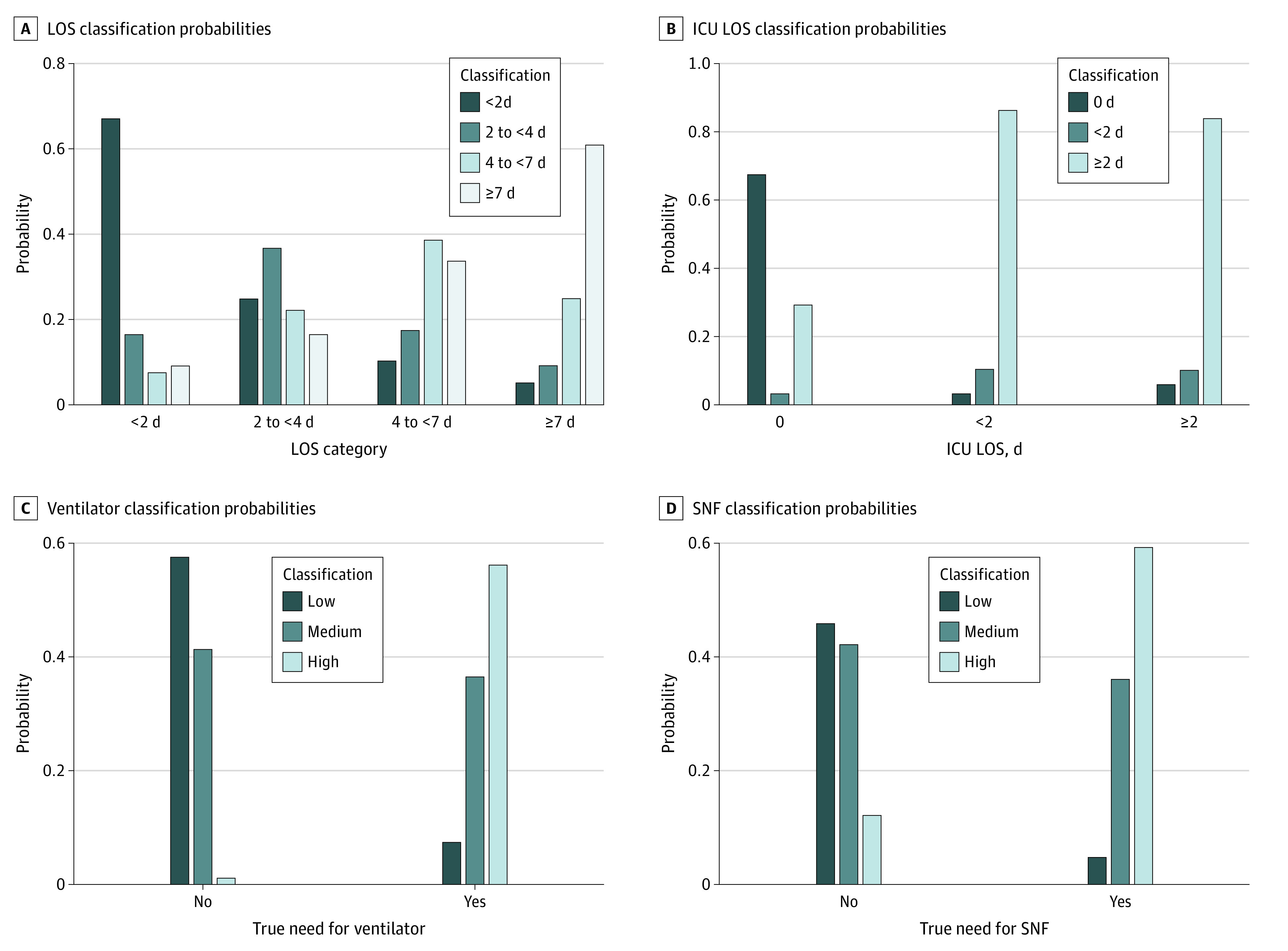
Performance of Each of the 4 Classification Models A, 786 of 1291 patients (60.9%) are categorized as having a long length of stay (LOS), while 3721 of 5561 patients (66.9%) are categorized as having a short LOS. B, 573 of 689 patients (83.2%) with a long intensive care unit (ICU) stay are correctly classified, as are 8052 of 11928 patients (67.5%) with no ICU stay. C, Of 529 patients who needed a ventilator, 503 (95.1%) are in the medium- or high-risk categories. D, Of 952 patients who will be discharged to a skilled nursing facility (SNF) 904 (94.9%) are in the medium- or high-risk categories.

Using the training data results, we created decision-rule thresholds for each of the outcomes based on sensitivity and positive predictive value (eFigures 2, 3, and 4 in the Supplement). For classifying LOS, 37121 of 5561 patients (66.9%) with a 0- to 2-day LOS and 786 of 1291 patients (60.9) with an LOS of 7 days or more were correctly classified (Figure 1A; eTable 3 in the Supplement). For ICU LOS, 2051 of 2141 patients (95.8%) with any ICU stay were classified as such. Moreover, the model correctly classified 23 8052 of 11 928 patients (67.5%) with no ICU visit and 573 of 689 patients(83.2%) with a long ICU stay. Only those with a short ICU stay tended to be misclassified (10% correct), reflective of both the poorer second-stage model and the desire for a decision rule with higher sensitivity (Figure 1B; eTable 2 in the Supplement). Of those needing a ventilator, 95% were predicted as being at high or medium risk, with the high-risk group having a positive predictive value of 70%. The negative predictive value of the low-risk group was more than 99% (Figure 1C; eTable 2 in the Supplement). Finally, for discharge to an SNF, we were able to generate a decision rule with a positive predictive value of 22% for the high-risk group and the desired 95% sensitivity for the combined medium- and high-risk groups (Figure 1D; eTable 2 in the Supplement) Additional performance metrics are presented in eFigures 5 and 6 in the Supplement.

### Relative Importance of Predictor Variables

To evaluate which factors were important predictors and what results to visualize in our dashboard, we examined the top 5 most important variables for each of the 5 models (Table 2). Patient age and the number of previous outpatient encounters, as well as specific service line and specialty division, were consistent predictors of higher resource utilization. We note that these are simply rankings of variables we considered important and are not associated with traditional metrics of statistical significance.

**Table 2.** Top Predictor Variables From Each of the Models

Outcome	Length of stay	Need for ICU	ICU length of stay	Need for ventilator	Discharge to SNF
Top predictor					
1	Age	Specialty	Age	Age	Procedure type: coronary artery bypass grafting
2	No. of previous hospital encounters	Service	No. of previous outpatient encounters	No. of previous outpatient encounters	Procedure type: endoscopic video of harvest vein bypass
3	No. of previous outpatient encounters	Procedure type: microsurgery	Specialty	Service	Procedure type: valve surgery
4	Specialty	Age	Service	BMI	History of cardiac surgery
5	Service	Procedure type: excise supratentorial brain tumor	BMI	No. of previous emergency encounters	Age

Abbreviations: BMI, body mass index; ICU, intensive care unit; SNF, skilled nursing facility.

### Dashboard

**Figure 2.**
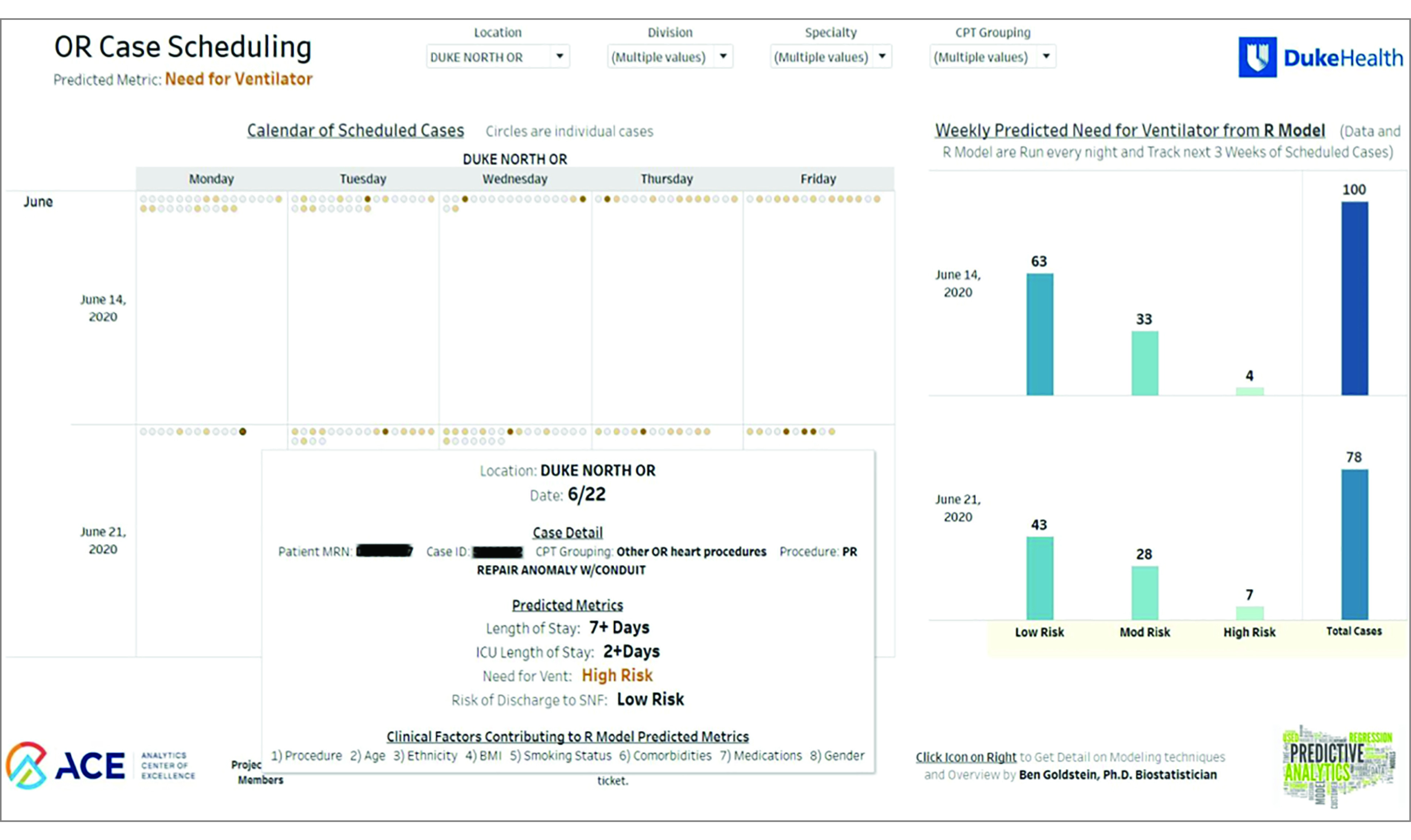
Screenshot of Calendar View of Dashboard The calendar view provides a look at the upcoming weeks and which cases are most risky. A user can click on an individual case to get more details. Each outcome has its own calendar view.

**Figure 3.**
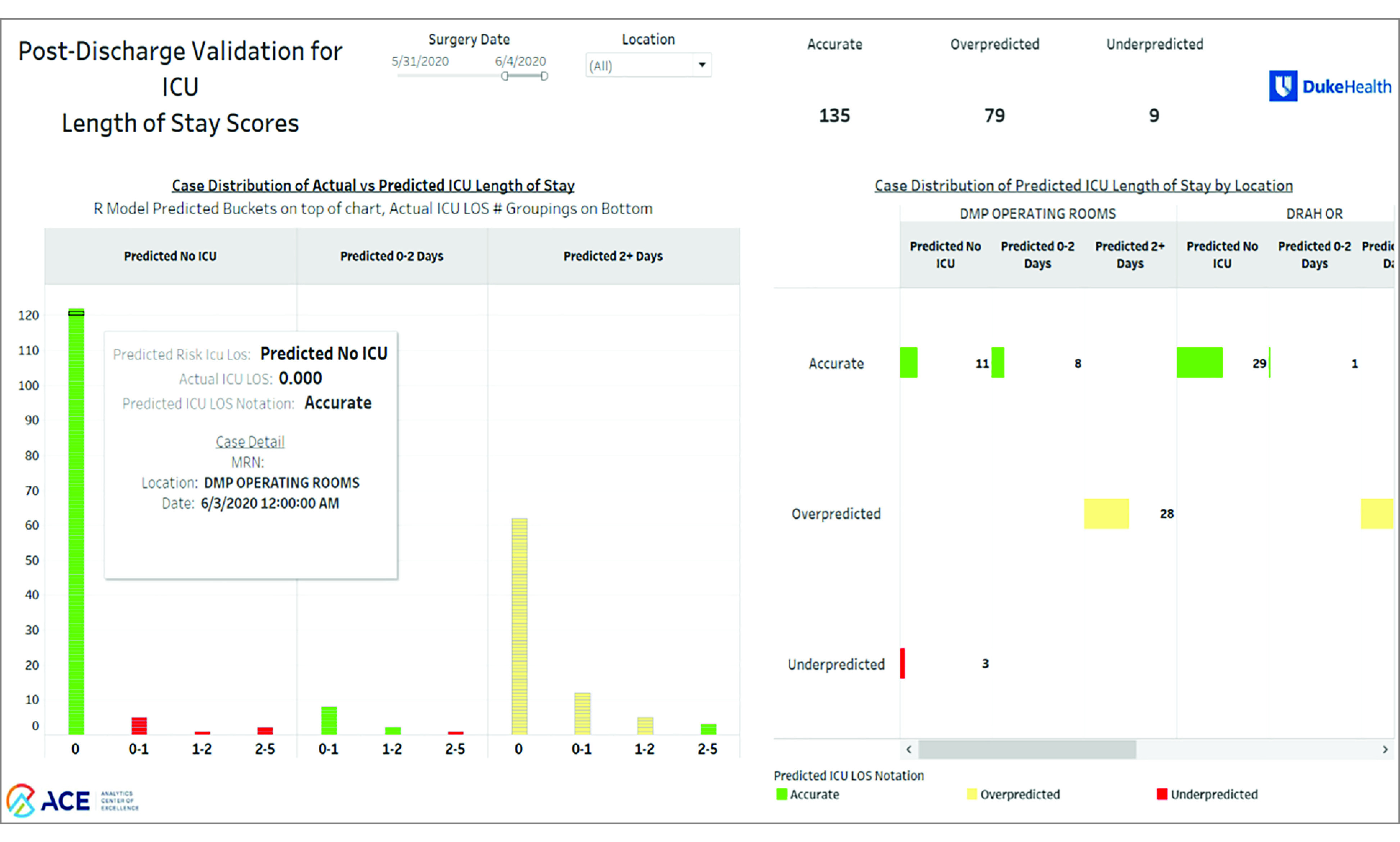
Screenshot of Monitoring View of Dashboard The monitoring view allows the user to observe how the predictive modeling has been performing. The green bars indicate correct predictions, the yellow bars indicate the overestimation of risk, and the red bars indicate the underestimation of risk. As reflected, the model was designed to overestimate vs underestimate risk.

We integrated the predictive model into a dashboard that shows the scheduled cases over the next month and that is refreshed every morning. Figure 2 shows the calendar view for one of the outcomes, LOS. eFigure 7 in the Supplement shows the primary landing page, and Figure 3 shows a model evaluation page that allows for a real-time, ongoing monitoring of the model’s performance. The interactive nature allows decision makers to assess subsamples based on week, location, service line, or procedure grouping.

## Discussion

To guide decisions around recommencing nonurgent surgical procedures, we produced a novel CDS tool that assesses the risk of high resource utilization for scheduled cases. Our tool identifies those at highest risk to avoiding exceeding hospital capacity. We integrated the CDS into a dashboard that is refreshed daily and provides a global assessment of upcoming cases. We believe that this tool, in conjunction with knowledge of currently available resources—including overall hospital and ICU beds, ventilator use, and area SNF capacity—will help leadership decide whether a case should proceed or be postponed.

Our CDS tool produces predictions of 4 outcomes: LOS, ICU LOS, need for ventilator, and need for discharge to an SNF. Overall, the models performed very well with AUCs in the training and testing data ranging from 0.76 to 0.93. However, instead of presenting predicting probabilities, we presented the predictions in risk thresholds with known performance characteristics. To prevent the underestimation of resource needs and the misclassification of high-risk patients as low-risk patients, we set the low-risk threshold to include less than 5% of those with resource needs. Our low-risk categories all had high negative predictive values (approximately 99%), allowing us to safely consider that those designated as low risk are in fact low risk.

The most predictive variables were demographic, service utilization, and procedural factors. Interestingly, clinical information was not among the top predictors—although it was still a part of the overall models. This highlights the ability to use easily retrievable information available at the time of scheduling to develop a strong CDS.

We established a data-flow process in which information on scheduled cases—including predicted resource utilization—are integrated into a dashboard that is refreshed each morning. While most traditional CDS presents information on 1 patient at a time, our display allows decision makers to examine the patient landscape more globally. The CDS tool is applicable across our 3 hospitals, all surgical specialties, and all patient age groups. While different specialties likely have different risk factors,^25^ a machine learning approach allows the model to find the appropriate degree of heterogeneity. In contrast, many prior investigations into resource utilization have been specialty- or procedure-specific investigations.^26,27,28^ Our integrated approach highlights how perioperative decision-making can be integrated into a hospital’s ultimate responsibility to public health in a time of critical shortages of resources due to community infectious risks.

### Tool Utilization and Further Developments

The CDS tool and associated dashboards were implemented on June 17, 2020. Owing to our local COVID-19 conditions, we have not had to make decisions regarding case prioritization. The tool has seen the greatest use by our case-management team, allowing them to do preplanning regarding who will be discharged to an SNF. We are also currently assessing how the tool can be used to do resource planning in a post–COVID-19 environment.

Overall, we view this as an ongoing learning process. Since launching the tool, we have evaluated alternative modeling approaches, such as specialty- and age-specific models. We have made modifications both speeding up the data flow process and amending aspects of the decision rule to better meet user needs. We created a model evaluation visual within an interactive visualization tool that allows the user to assess the performance of the risk model. This allows us—as the tool developers—to monitor performance and allows tool users to develop confidence in the quality of the CDS. Finally, we are investigating ways to more tightly align this tool with other dashboards indicating ICU census and local COVID-19 conditions.

### Limitations

The models described herein and the CDS they underpin provide both a proof of concept and a blueprint for other institutions. However, our approach is not without limitations. First, the specific model was constructed and validated using data generated in a single hospital system. This limits its external validity, although we hypothesize that this approach—using similar predictor variables—should perform comparably in similar settings (ie, for tertiary academic medical centers). Moreover, our analytic framework cannot provide insight into how to optimally integrate current resource availability with future resource needs from a surgical standpoint. The former is subject to various other factors, including community, day of week, and seasonal variation. To achieve precision that is sufficiently adequate for day-to-day decision-making, we determined that clinically meaningful groupings (eg, extended hospital stay and short vs long ICU stays) were preferable to predicted probabilities. As such, while the underlying predictions are fundamentally quantitative, our formulation of outputs is designed to augment and inform, but not replace, the surgeon’s decision to perform an operation. Therefore, this type of tool supports a system for determining whether hospital resources are at risk of being overwhelmed on any given day or week; it is to be used together with the background COVID-19 rate in the community, a current inpatient and ICU census, an understanding of deferred cases, and the medical necessity of any 1 patient. This highlights the complexity of making such decisions and the need for better integration of various CDS tools. Finally, our model is only as precise as the information interpretable from the medical record. Although patient-level data are subject to misspecification and miscoding, we have devised an approach that is iterative so that predictions can be updated with new inputs as decisions are made and acted on each day forward.

## Conclusions

The framework that we present for building a formalized CDS tool for surgical resource utilization, in conjunction with the workflow of integrating EHR data with a dashboard, is highly replicable. As institutions attempt to chart a safe and sustainable path to a new normal, this work illustrates how surgical teams and hospital leadership can do so in a data-driven way, generating a learning health care environment.

## Notes

Supplement. **eFigure 1.** Distribution of Length of Stay (a) and ICU Length of Stay (b) **eTable 1.** Predictor Variables Used **eAppendix.** Methods **eFigure 2.** Creating a Decision Rule for ICU Length of Stay **eFigure 3.** Creating Decision Rule for Need for Ventilator **eFigure 4.** Creating Decision Rule for Discharge to SNF **eTable 2**. Performance of Decision Rules **eTable 3**. Length of Stay Classifications **eTable 4.** Performance in Training and Testing Data **eFigure 5.** Area Under the Receiver Operator Characteristic **eFigure 6.** Area under the Precision-Recall Curve **eFigure 7.** Executive Summary Landing Page for the Tableau Dashboard
