# Supplementary material for: Development and Performance of a Clinical Decision Support Tool to Inform Resource Utilization for Elective Operations
Source: JAMA Netw Open. 2020 Nov 2;3(11):e2023547. doi: 10.1001/jamanetworkopen.2020.23547 (PMC7607444; doi:10.1001/jamanetworkopen.2020.23547)
Supplement: Supplement. — eFigure 1. Distribution of Length of Stay (a) and ICU Length of Stay (b) eTable 1. Predictor Variables Used eAppendix. Methods eFigure 2. Creating a Decision Rule for ICU Length of Stay eFigure 3. Creating Decision Rule for Need for Ventilator eFigure 4. Creating Decision Rule for Discharge to SNF eTable 2. Performance of Decision Rules eTable 3. Length of Stay Classifications eTable 4. Performance in Training and Testing Data eFigure 5. Area Under the Receiver Operator Characteristic eFigure 6. Area under the Precision-Recall Curve eFigure 7. Executive Summary Landing Page for the Tableau Dashboard [file jamanetwopen-e2023547-s001.pdf]

Supplemental Online Content

Goldstein BA, Cerullo M, Krishnamoorthy V, et al. Development and performance of a clinical decision support tool to inform resource utilization for elective operations. *JAMA Netw Open*. 2020;3(11):e2023547. doi:10.1001/jamanetworkopen.2020.23547

eFigure 1. Distribution of Length of Stay (a) and ICU Length of Stay (b)

eTable 1. Predictor Variables Used

eAppendix. Methods

eFigure 2. Creating a Decision Rule for ICU Length of Stay

eFigure 3. Creating Decision Rule for Need for Ventilator

eFigure 4. Creating Decision Rule for Discharge to SNF

eTable 2. Performance of Decision Rules

eTable 3. Length of Stay Classifications

eTable 4. Performance in Training and Testing Data

eFigure 5. Area Under the Receiver Operator Characteristic

eFigure 6. Area under the Precision-Recall Curve

eFigure 7. Executive Summary Landing Page for the Tableau Dashboard

This supplemental material has been provided by the authors to give readers additional information about their work.

© 2020 Goldstein BA et al. *JAMA Network Open.*

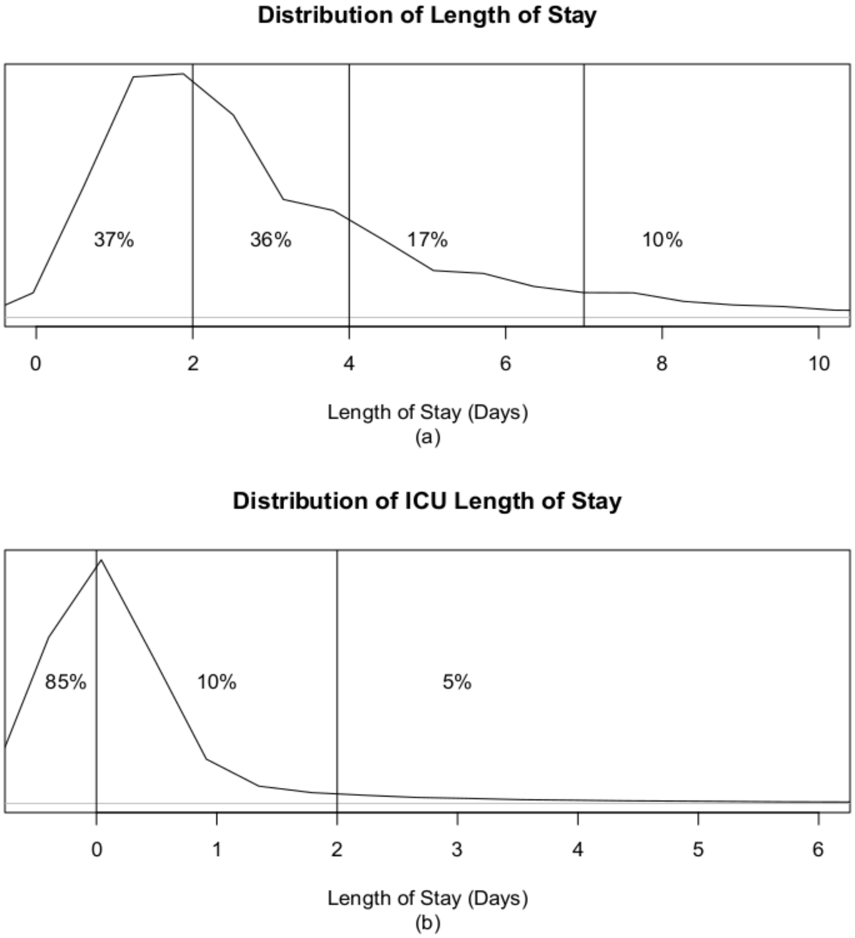

Figure 1. Distribution of length of stay (a) and ICU length of stay (b). Cut-points indicate where categorizations were made.

© 2020 Goldstein BA et al. *JAMA Network Open.*

eTable 1: Predictor Variables Used

VARIABLE	NOTES
**Demographics**	
Age	
Sex	
Race	Non-hispanic white, non-hispanic black, hispanic, other
Smoking Status	Ever/Never
BMI	Categorized as normal, overweight, obese, underweight, missing
**Service Utilization**	Based on past year
Number of previous outpatient encounters	
Number of previous inpatient encounters	
Number of previous emergency encounters	
**Procedure Information**	
CPT Code	Procedures were grouped according to the Agency for Healthcare Research and Quality (AHRQ) clinical classification software (CCS) adaptation for use with current procedural terminology (CPT) codes. Procedures that were performed fewer than 75 times in this period (< 2x/mth) were grouped into an “other” category, resulting in 150 unique procedures.
Service Line	Indicators for 12 different service lines. Defined based on service lines that appeared at least 25 times in the historic data.
Specialty	Indicators for 21 different specialties. Defined based on specialties that appeared at least 25 times in the historic data.
OR Type of Procedure	Categorized as “Major”, “Moderate”, “Minor” and “None”
**Comorbidities**	Based off of EPIC Groupers past 2 years
Diabetes	
COPD	
Congestive Heart Failure	
Myocardial Infarction	
Hypertension	
Peripheral Vascular Disease	
CerebroVascular Accident - Transient Ischemic Attack	
Atrial Fibrillation	
Atherosclerotic Cardiovascular Disease	
Coronary Artery Disease	
Cardiovascular Disease	
Renal Diabetes	

© 2020 Goldstein BA et al. *JAMA Network Open.*

End Stage Renal Disease	
Pulmonary Hypertension	
Stent Placement	
Cardiac Surgery	
**Medication**	Based off EPIC groupers in the past 30 days
Hypertension Medications	
ACE Inhibitors	
ARBs	
Beta Blockers	
Calcium Channel Blockers	
Digoxin	
Diuretics	
Nitrates	
Statins	
Opioids	
Oral Diabetic	
Anti-Coagulants	
Anti-Platetlets	
Anti-Arrhythmics	
Psychiatric Medications	
Insulin	
Heart Failure Medication	

eAppendix. Methods.

The Random Forests Algorithm

RF is a machine learning algorithm that combines a series of decision trees into a single classifier. It is well suited for handling variables with non-linear effects and possible interactions. Moreover, it is well suited for predicting multi-class outcomes. Like many machine learning algorithms, RF does not produce traditional coefficients that can be interpreted as the degree of association between independent and dependent variables; however, RF produces a variable importance measure, which indicates the extent that individual predictor variables drive the final prediction. Finally, RF performs an internal cross-validation to assist in the selection of tuning parameters and assessment of model performance. We used this feature to evaluate its performance on the training data.

© 2020 Goldstein BA et al. *JAMA Network Open.*

Setting Model Classification Threshold

We set model classification as follows. For overall LOS, the outcome class (i.e., the range of days) was defined as the class for which the predicted risk resulted in the largest increase in relative risk. For ICU LOS, we defined a sensitivity principle where 95% of those who required the ICU would be within the short and long stay class. If a patient passed this threshold in the first model, they were moved into the second to predict length of ICU stay. For this model we similarly chose a 95% sensitivity threshold where 95% of those with a long stay would be classified within the long stay class. For the two binary classification models (need for ventilator and SNF), we created groupings of low-, medium-, and high-risk. For the low-risk category threshold, we set it to have a sensitivity of 5%, meaning that 95% of true cases would be contained within the medium- and high-risk categories. For the high-risk category, we examined the predicted risk distribution and chose what we felt would be the best cut-point based on the positive predictive value (PPV) and prevalence of the outcome.

© 2020 Goldstein BA et al. *JAMA Network Open.*

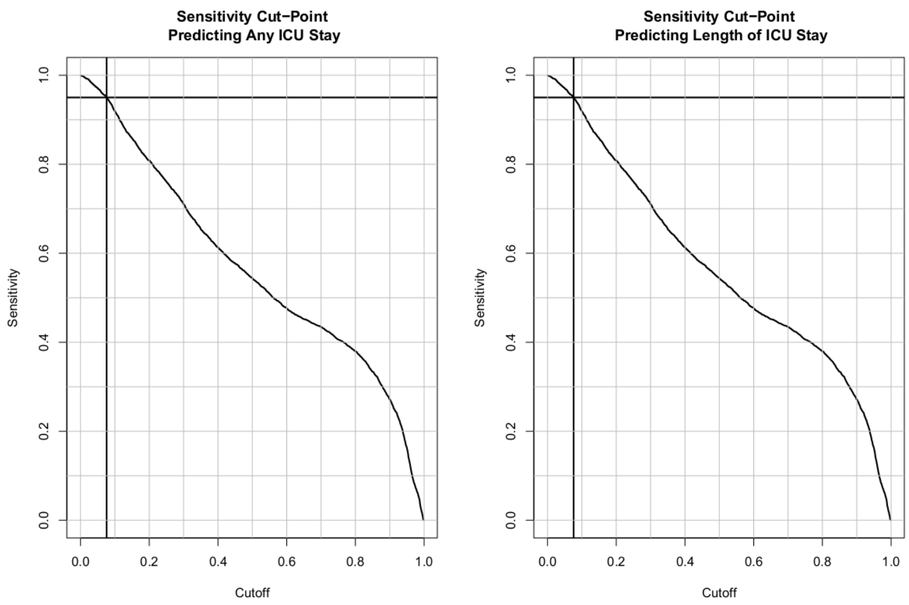

eFigure 2. Creating a decision rule for ICU Length of Stay. There is a 95% sensitivity for Any ICU visit. There is a 95% sensitivity for capturing long ICU Length of Stay.

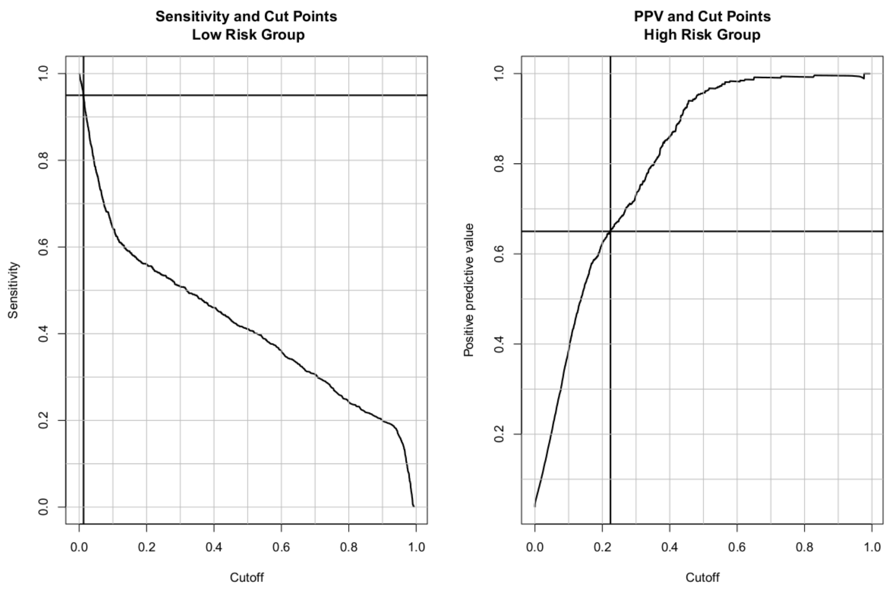

eFigure 3. Creating decision rule for need for ventilator. The low/medium risk group generates a sensitivity of 95%. The medium/high risk grouping generates a positive predictive value 65%.

© 2020 Goldstein BA et al. *JAMA Network Open.*

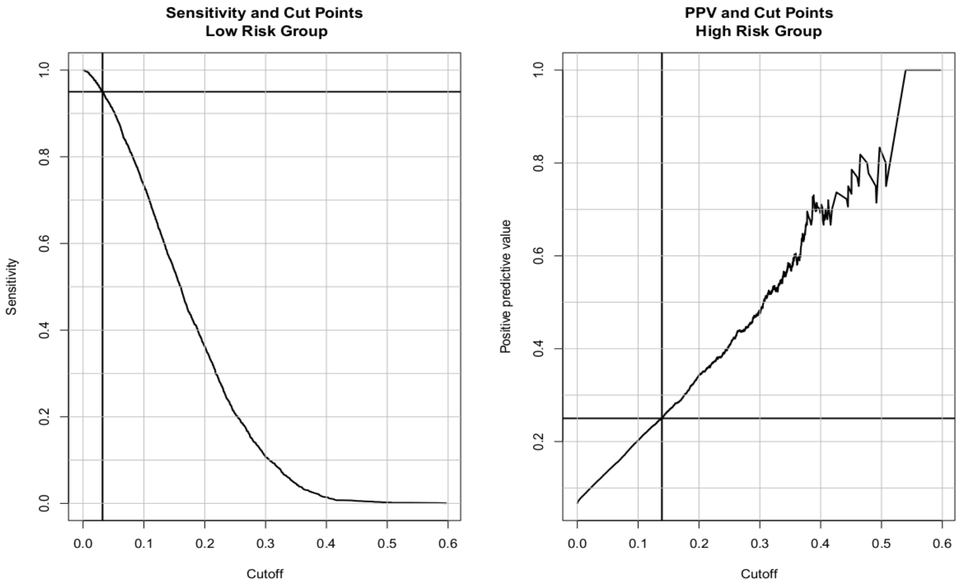

eFigure 4. Creating decision rule for discharge to SNF. The low/medium risk group generates a sensitivity of 95%. The medium/high risk grouping generates a positive predictive value 25%.

eTable 2. Performance of decision rules

A. Performance of decision rule for need for any ICU stay in Test Data

	**Need Any ICU**	**2+ Day ICU Stay**
**% of Sample Need**	45%	40%
**Sensitivity**	0.95	0.83
**Specificity**	0.65	0.62
**Positive Predictive Value**	0.33	0.11
**Negative Predictive Value**	0.99	0.98

B. Performance of decision rule for need for ventilator in Test Data

	**Low Risk**	**Medium Risk**	**High Risk**
**% of Sample**	50%	47%	3%
**Sensitivity**	0.05	0.40	0.55
**Specificity**	0.52	0.47	0.01
**Positive Predictive Value**	< 0.01	0.03	0.70
**Negative Predictive Value**	> 0.99	0.97	0.30

C. Performance of Decision Rule for Discharge to SNF in Test Data

	**Low Risk**	**Medium Risk**	**High Risk**
**% of Sample**	40%	44%	16%
**Sensitivity**	0.05	0.40	0.55
**Specificity**	0.42	0.44	0.13

© 2020 Goldstein BA et al. *JAMA Network Open.*

**Positive Predictive Value**	0.01	0.06	0.22
**Negative Predictive Value**	0.99	0.94	0.78

eTable 3. Length of Stay Classifications

A. Length of Stay Classifications—Training Data

Actual/Predicted	0 – 2 Days	2 – 4 Days	4 – 7 Days	7+ Days
**0 – 2 Days**	7471	1811	857	980
**2 – 4 Days**	2511	3516	2162	1588
**4 – 7 Days**	431	875	1763	1487
**7+ Days**	168	272	637	1610

B. Length of Stay Classifications—Testing Data

Actual/Predicted	0 – 2 Days	2 – 4 Days	4 – 7 Days	7+ Days
**0 – 2 Days**	3721	912	424	504
**2 – 4 Days**	1220	1806	1090	809
**4 – 7 Days**	237	400	884	771
**7+ Days**	68	116	321	786

Contextualization of Performance Metrics

While many factors dictate the clinical utility of a model, there are certain rubrics for AUROC and Calibration. Since AUPRC is determined by prevelance of the outcome, such rubric do not exist. In general, AUROC > 0.9 is extremely good, AUROC > 0.8 is very good, AUROC > 0.7 is acceptable and AUROC < 0.7 is not clinically useful. For calibration, we desire the calibration slope to be as close to 1 as possible.

eTable 4. Performance in Training and Testing Data

	ICU: Yes/No	ICU: Long/Short	VENT	SNF
**AUROC - Train**	0.93	0.76	0.92	0.84
**AUPRC - TRAIN**	0.81	0.64	0.63	0.3
**Calibration - TRAIN**	1.30	1.22	1.15	1.33
**AUROC - TEST**	0.94	---*	0.91	0.85
**AUPRC - TEST**	0.82	---*	0.64	0.32
**Calibration - TEST**	1.34	---*	1.18	1.40

* Predictive performance was not assessed on the test data because not everyone who made it to stage two was within truly within one of the two classes

© 2020 Goldstein BA et al. *JAMA Network Open.*

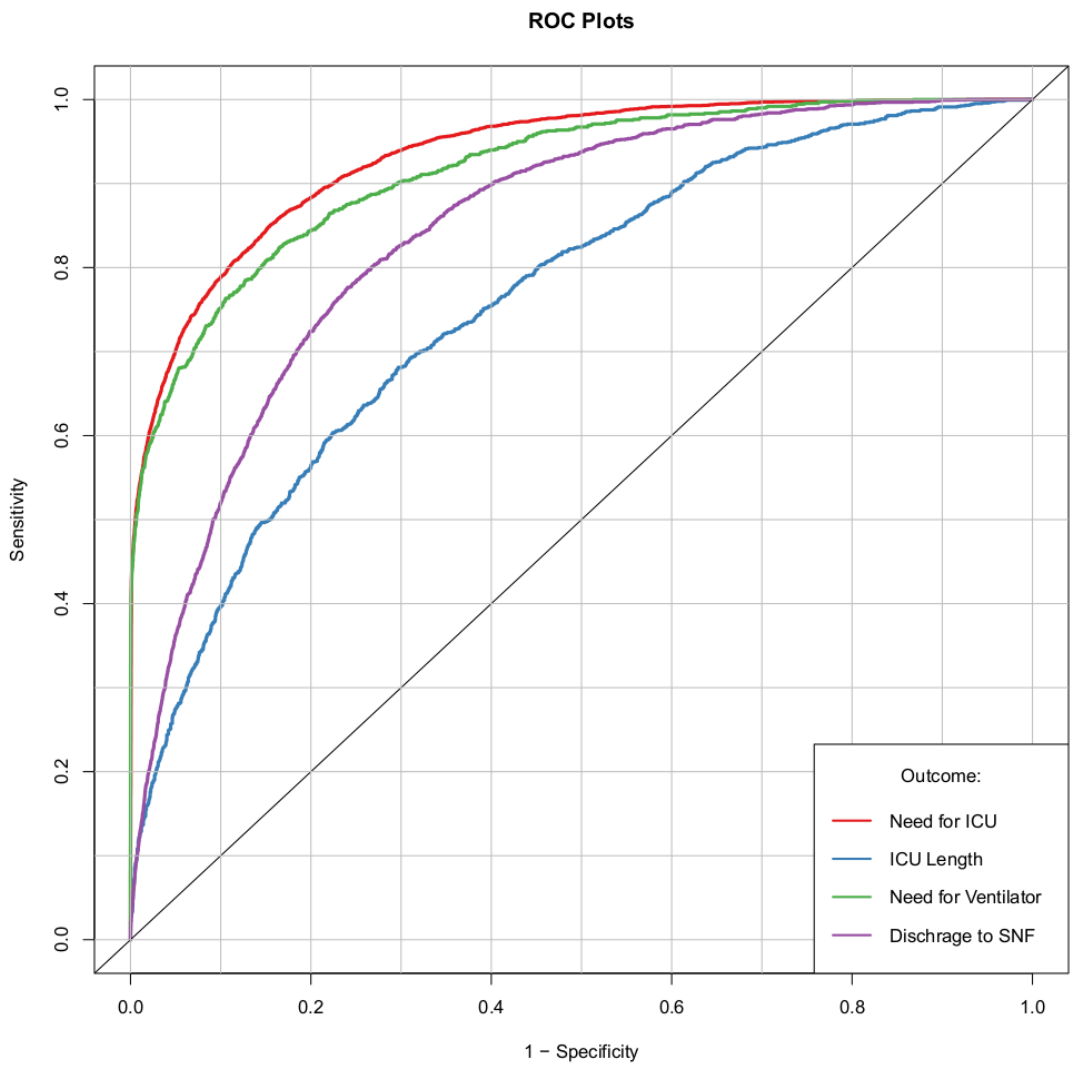

eFigure 5. Area Under the Receiver Operator Characteristic. Need for ICU and Need for Ventilator have the best discrimination

© 2020 Goldstein BA et al. *JAMA Network Open.*

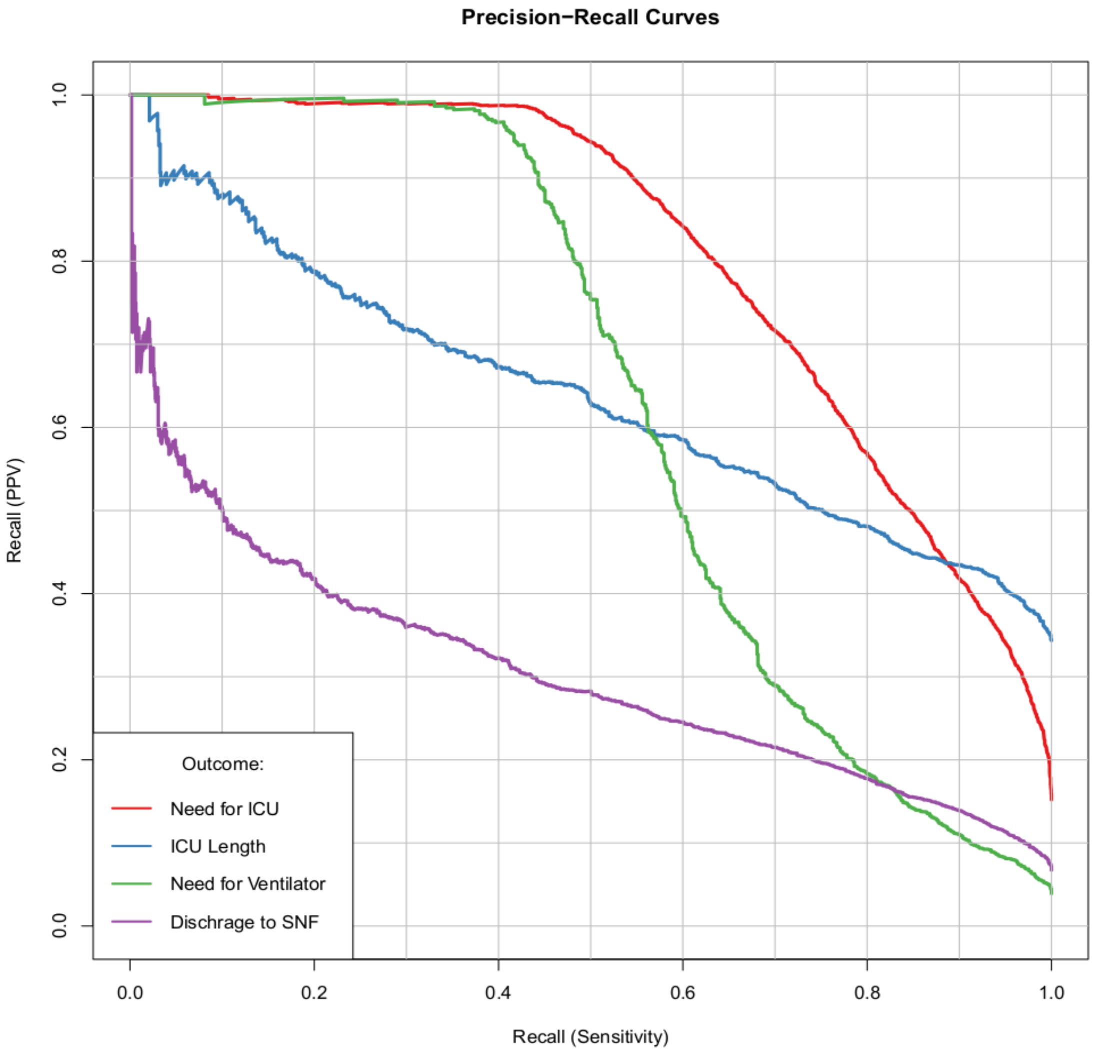

eFigure 6. Area under the Precision-Recall Curve. Need for ICU and Need for Ventilator show the best performance

© 2020 Goldstein BA et al. *JAMA Network Open.*

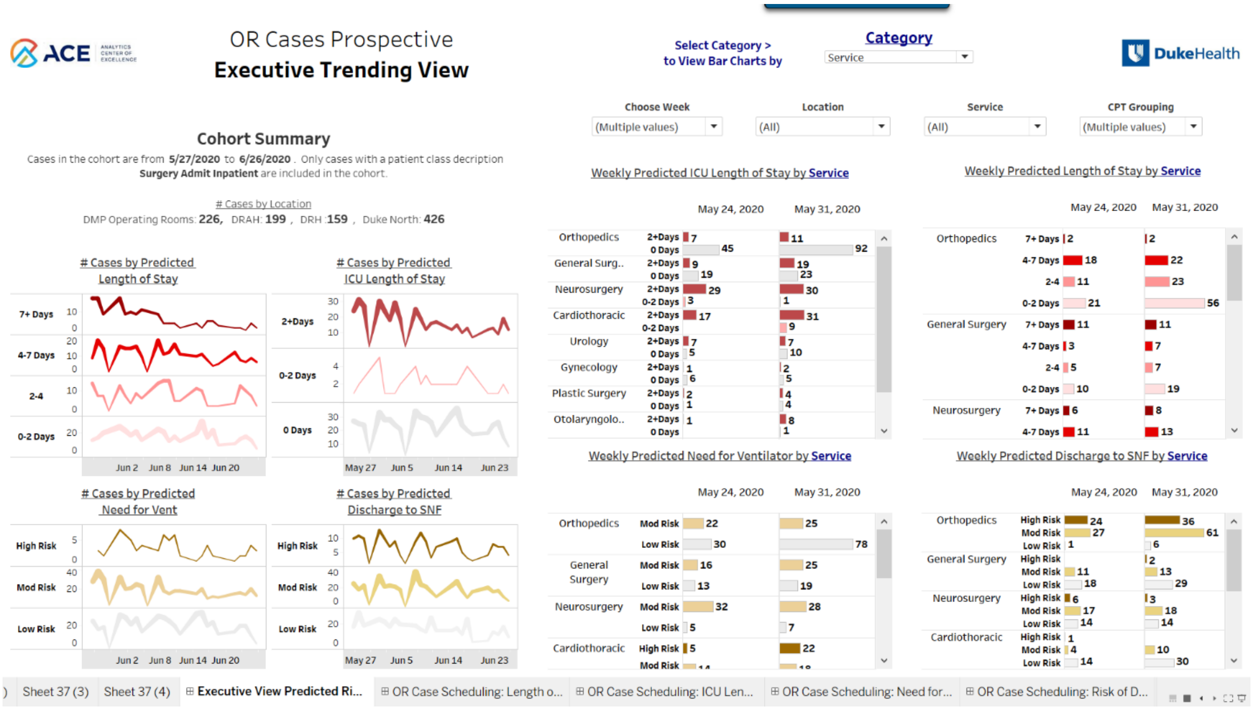

eFigure 7. Executive summary landing page for the Tableau dashboard. Shows overall assessment for upcoming cases as well as options for specialty and case specific assessments.

© 2020 Goldstein BA et al. *JAMA Network Open.*
